# Supplementary material for: Patterns of Intron Gain and Loss in Fungi
Source: PLoS Biol. 2004 Nov 30;2(12):e422. doi: 10.1371/journal.pbio.0020422 (PMC532390; doi:10.1371/journal.pbio.0020422)
Supplement: Table S1 — Also available at http://genes.mit.edu/NielsenEtAl/. (4.3 MB ZIP). [file pbio.0020422.st001.zip › NielsenEtAl/html/598.html]

AN0403.1.NCU06061.1.MG08290.1.FG04335.1


```
 CLUSTAL W (1.82) Multiple Sequence Alignments - Introns Inserted


Sequence 1: MG08290.1	427 aa
Sequence 2: FG04335.1	527 aa
Sequence 3: NCU06061.1	433 aa
Sequence 4: AN0403.1	534 aa
Alignment Length: 576 aa
Number Identitical Residues: 129 aa
Alignment Score (without introns) 7529


MG08290.1 	---------------------------------~--------------------------
NCU06061.1	---------------------------------~--------------------------
FG04335.1 	MRNWGLVMTETREMNPCSKKEAGLGPMLRAFSR0---LMIISRVSTKLPGRLFKSPNASY
AN0403.1  	MLTSRLPVVQYRVRPRHLARYFWKNLLLQPQTR~PLSLLQTPRHQTPRTGANLQRCRYVH
          	  .      .              .    . :  . :    .  .:  ..   .      

MG08290.1 	-------------------------MTVSAQS---~-----------------------S
NCU06061.1	----------------------MGSIAVDDATLS-~----------------------RP
FG04335.1 	TQAKPPFASRASLPTLVALNHRISATSVSTTSRLP~SRKRPIAHRLKGFATMGSLPETSN
AN0403.1  	QSYRNVSTTGQTPESLKSQAPQTPGRGFAVKSLYR2-------------------FEQAQ
          	 .     ::  :  :  :      .  .   :                        .   

MG08290.1 	PYKVLVVGGSYAGISAVLNLIDLCTGNSPRCGARVDDSEAS-------KIPIDVDVTIVD
NCU06061.1	PFKVLVVGGSYGGLSAALNLQDICSGRAARCGPDAKKAALDGTSAAHDGPQFAVDITVVD
FG04335.1 	AVRVLILGGCYGGLSAAVNLLDLSQGYSPRMNSEPYTHHPN-------LPTFNIEITIVD
AN0403.1  	PFKVLILGGSYAGLAAALNLVDLCHGRRHRFSITEGDNGTG--------KRIPVQVTIVD
          	. :**::**.*.*::*.:** *:. *   * .        .          : :::*:**

MG08290.1 	ERDGFF1HIIGAPLALASSSFAEKAWVKYSDIAALQRP--NIRIIQGTAKTVDPTTKTAT
NCU06061.1	ERDGFY1HLIGTPLAFADESYAEKCWVKYEDVPALQQSPNNIRILRGSVQSIDQERKIAK
FG04335.1 	QRDGYY1HLIGSPMALADSAFSKKNWVKYSDIPGLKDP--RINIIQGSVTGVDPASKKAT
AN0403.1  	PRDGYY1HLIGQPLALSSQEFAKSFWIKYTDIPALQTP--EIRCVQGSIDSLDCTAKTAT
          	 ***:: *:** *:*::.. :::. *:** *:..*: .  .*. ::*:   :*   * *.

MG08290.1 	ITRTDPAAAAQPSSDEIIVYDFLVAASGLRRVWPVVPQSLRRKQYLLETGSHVQAVSGAR
NCU06061.1	VLG-----SENGPGPIELKYDYLIAAAGLRRVFPVVPQSLLRKQFLFEAGDHIQAATANR
FG04335.1 	IS------AHLTEEKSTLEYDYLVAATGLRRVWPVVPQSLTRKQYLFEAENHINAVQNAK
AN0403.1  	IAT--------ESGAVQEKYDYLIACTGLRREFPSAPRSLTRETYLAETAENLANIRGAE
          	:                  **:*:*.:**** :* .*:** *: :* *: .::      .

MG08290.1 	NGVLVVGGG~AVGIEMAAELKLIQPHVRVTLAHSRDRLLSSEALSDECKDVALGLLREAG
NCU06061.1	HGVAVVGGG1AVGIEMAAELKLVCPDLKVTLIHSRDKLLSAEPLPEEVKDRSLELVHEAG
FG04335.1 	HGVVVVGGG~AVGIEMAAELKMVKPHLNVTLVHSRDKLLSSEGLPDETKDVALELLREAG
AN0403.1  	KGVAVIGGG1AVGIEIAAECKMLHPDTPVTLIHSRSSLLSSEPLPAEFASKALEALRGNS
          	:** *:*** *****:*** *:: *.  *** ***. ***:* *. *  . :*  ::  .

MG08290.1 	VEVLMGHRIAEQVEVTTEDGGKAVEARFENGHRLVVDNVIMAISKSVPTS-SYLPVEALD
NCU06061.1	VEVLMSHRVDRTEEFVDN-GHKAYKVYFTNGHTLIADSVIMAISRSVPST-TFLPRDVLN
FG04335.1 	VEVLMNHRLASKNKLETTDGSEKYDVEFTNGHKMSASVVIMAISRSVPTT-TYLPTSALD
AN0403.1  	VNVILGARVTS---ITEKDSAQNQTLTLTTNETLTASHVINAVSRYTPTAPSFLPASVCD
          	*:*::. *:     .   .. :     : ... : .. ** *:*: .*::.::** .. :

MG08290.1 	VEGYVKIQPS2IAFPPS----IPNAEYHYAPGDLAHWSGIKRCGGAMHMGFCAATNMHQL
NCU06061.1	EEGYVKIQPS2LHFPSE----SPNSDDHLAIGDLVKWSGIKRCGGAMHMGYLAANNIHMR
FG04335.1 	EEGFVKIKPN2LQFEQG----TPNAESHYAAGDITRWPGIKRCGGAMHQGHYVALNIHQN
AN0403.1  	EHGYIRITPT~LEFPSDSVLPASVAGDHYAAGDVARWSGVKRAGAAMHQGHYAARNIHQK
          	 .*:::* *. : *   :  . . :  * * **:.:*.*:**.*.*** *. .* *:*  

MG08290.1 	MLQEMSAGQHIPKPIKLSEFPPMIGLAVGKKAVSYSPDAGTNSGEDVAEMFFGNDLGFSI
NCU06061.1	MKQAVSG--TEPTYLELDEIPPMIGLAVGKKAVAYWPTNGVSSGEDVNKLFFGDDLGFDI
FG04335.1 	ILSQRAG--HIPSYKEIAVYPPVIGLAVGKKAVASSPDTGTTAGEDVLQSCFRDDLGWTI
AN0403.1  	LMQRVYG--TTPEFVRLDEVEPGMGLAVGKRAVAYFPSMGLSEGEETREMFFQGDLGFMI
          	: .   .    *   .:    * :******:**:  *  * . **:. :  * .***: *

MG08290.1 	1CWNYLRLGG------------------------DKETEKI-
NCU06061.1	1CWNHLRLGG------------------------YDVVKA--
FG04335.1 	1CWNYMQLGG------------------------RKTDEAKA
AN0403.1  	1CYKWMQLGTPMPPPVADAPASTPEDKPVNVATTSRTVEAAA
          	 *:: ::** . ... :.:.:::..... . :::    :  :
```
